# Severe Genotype, Pancreatic Insufficiency and Low Dose of Pancreatic Enzymes Associate with Abnormal Serum Sterol Profile in Cystic Fibrosis

**DOI:** 10.3390/biom11020313

**Published:** 2021-02-19

**Authors:** Sławomira Drzymała-Czyż, Patrycja Krzyżanowska-Jankowska, Krzysztof Dziedzic, Aleksandra Lisowska, Szymon Kurek, Joanna Goździk-Spychalska, Victoria Kononets, Dagmara Woźniak, Edyta Mądry, Jarosław Walkowiak

**Affiliations:** 1 Department of Pediatric Gastroenterology and Metabolic Diseases, Poznan University of Medical Sciences, 60-572 Poznań, Poland; pkrzyzanowska@ump.edu.pl (P.K.-J.); dziedzic@up.poznan.pl (K.D.); alisowska@ump.edu.pl (A.L.); skurek@ump.edu.pl (S.K.); jarwalk@ump.edu.pl (J.W.); 2 Department of Bromatology, Poznan University of Medical Sciences, 60-354 Poznań, Poland; dagmara.wozniak94@gmail.com; 3 Department of Food Science and Nutrition, Institute of Food Technology of Plant Origin, Poznań University of Life Sciences, 60-637 Poznań, Poland; 4 Department of Pulmonology, Allergology and Respiratory Oncology, Poznan University of Medical Sciences, 60-569 Poznań, Poland; jogoz@ump.edu.pl; 5 Department of Natural Sciences Disciplines, West Kazakhstan Marat Ospanov Medical University, Aktobe 030012, Kazakhstan; micropaleontolog@yandex.kz; 6 Department of Physiology, Poznan University of Medical Sciences, 61-781 Poznań, Poland; emadry@ump.edu.pl

**Keywords:** campesterol, β-sitosterol, stigmasterol, cholesterol, lathosterol

## Abstract

Background: Several factors could lead to lipid disturbances observed in cystic fibrosis (CF). This study aimed to assess sterol homeostasis in CF and define potential exogenous and endogenous determinants of lipid dysregulation. Methods: The study involved 55 CF patients and 45 healthy subjects (HS). Sterol concentrations (μg/dL) were measured by gas chromatography/mass spectrometry. CF was characterised by lung function, pancreatic status, liver disease and diabetes coexistence, *Pseudomonas aeruginosa* colonisation and BMI. *CFTR* genotypes were classified as severe or other. Results: Campesterol and β-sitosterol concentrations were lower (*p* = 0.0028 and *p* < 0.0001, respectively) and lathosterol levels (reflecting endogenous cholesterol biosynthesis) were higher (*p* = 0.0016) in CF patients than in HS. Campesterol and β-sitosterol concentrations were lower in patients with a severe *CFTR* genotype, pancreatic insufficiency and lower pancreatic enzyme dose (lipase units/gram of fat). In multiple regression analyses, β-sitosterol and campesterol concentrations were predicted by genotype and pancreatic insufficiency, whereas cholesterol and its fractions were predicted by phytosterol concentrations, age, dose of pancreatic enzymes, nutritional status and genotype. Conclusions: Independent determinants of lipid status suggest that malabsorption and pancreatic enzyme supplementation play a significant role in sterol abnormalities. The measurement of campesterol and β-sitosterol concentrations in CF patients may serve for the assessment of the effectiveness of pancreatic enzyme replacement therapy and/or compliance, but further research is required.

## 1. Introduction

Cystic fibrosis (CF) is the most common genetic disease inherited in an autosomal recessive manner caused by mutations in the *CFTR* (*CF transmembrane conductance regulator*) gene located on the long arm of the seventh chromosome. The product of this gene is the CFTR protein present on the apical surface of the epithelial cells of the respiratory system, pancreas, intestine and sweat glands, where it acts as a chloride (Cl) channel [1,2]. The loss of functional CFTR Cl^−^ channels disrupts Cl^−^ transport across epithelia, which contributes to the production of thick, sticky mucus in secretory organs. CF involves the coexistence of pathological changes in various systems, including the liver and bile ducts [3,4,5], which directly affect fat metabolism. Lipid disorders in patients with CF have become a current topic of research; however, the causes of CF-related hypocholesterolemia remain unknown [6,7,8]. Studies conducted in animals and in vivo models with CF cells indicate that *CFTR* mutations affect the displacement of cholesterol to the cell membrane; in turn, cholesterol exhaustion leads to increased de novo cholesterol synthesis to restore the membrane content [9,10].

The observed lipid disturbances can be explained, at least in part, by the high fat intake recommended in CF and coexisting cholestasis [11,12]. Among the most important sources of fat in the CF diet are plant oils rich in phytosterols (such as campesterol, β-sitosterol, stigmasterol) [13], which regulate the circulation of cholesterol and lower its concentration in the blood. Plant sterols inhibit the absorption of cholesterol and can displace cholesterol from micelles because they are more hydrophobic than cholesterol and have a higher affinity for these micelles. Decreased cholesterol absorption results in increased endogenous biosynthesis. Physiologically, the excess of cholesterol produced is excreted with the bile and then reabsorbed into the bloodstream [14,15]. However, high phytosterol consumption can hinder reverse absorption and, at the same time, increase endogenous cholesterol production [16,17]. Simultaneous determination of plant sterols’ concentrations and the estimation of their consumption in patients with CF may help to understand the impact of nutrition on the observed fat disturbances.

Only two studies conducted by Gelzo et al. have assessed the total sterol profile in CF patients. In the first small study (*n* = 26), the authors suggested that sterol absorption is reduced and synthesis is increased in CF [18]. In the second study, the sterol profile was analysed in CF patients with pancreatic sufficiency and insufficiency [19], with lower cholesterol and phytosterol levels observed in pancreatic-insufficient patients. However, nutrition and enzyme therapy were not considered. Moreover, multiple analyses were not performed, which disenables the reliable assessment of lipid status in CF.

Our study is the first to define the exogenous and endogenous determinants of sterol abnormalities in CF patients using multiple regression analysis, simultaneously taking into account dietary sterol intake and pancreatic enzyme replacement therapy.

## 2. Material and Methods

### 2.1. Patients

The study comprised 55 CF patients (33 female, 22 male) aged 16 to 50 years. The inclusion criteria for CF patients were CF diagnosis compatible with the CF Foundation guidelines [20] and age ≥16 years. The exclusion criteria included pregnancy, non-CF gastrointestinal diseases with maldigestion and malabsorption, liver transplantation, other severe systemic diseases (neoplasms, endocrinopathies, liver cirrhosis) and lung or/and liver transplantation. The control group included 45 young adults (26 female, 19 male) aged 18 to 34 years. HS were excluded if they had a systemic/chronic/severe disease or a family history of hypercholesterolemia, or if they followed a restricted diet (e.g., any type of vegetarian diet). 

The body weight and height of all participants were measured, and BMI (body mass index, kg/m^2^) was calculated. Forty-eight CF patients were pancreatic-insufficient (faecal elastase-1 concentrations < 100 μg/g) [21,22] and took pancreatic enzymes. Individual CF characteristics were assessed (liver disease, diabetes, chronic and/or intermittent colonisation by *Pseudomonas aeruginosa*, forced expiratory volume in one second (FEV1) [23,24]) as well as genotypes, which were as follows: F508del/F508del (*n* = 17), F508del/3849 + 10kbC > T (*n* = 6), F508del/W1282x (*n* = 2), F508del/CFTRdel2,3(21kb) (*n* = 3), F508del/2143delT (*n* = 1), F508del/3600 + 2insT (*n* = 1), F508del/R352Q (*n* = 1), F508del/G551D (*n* = 1), F508del/N1303K (*n* = 1), F508del/3121-2A > G (*n* = 1), F508del/2183AA-G (*n* = 3), F508del/R851X (*n* = 1), F508del/1717-1G- > A (*n* = 1), F508del/- (*n* = 5), 3849 + 10kbC > T/3849 + 10kbC > T (*n* = 2), 3849 + 10kbC > T/dele2,3(21kb) (*n* = 1), 3849 + 10kbC > T/3600 + 1G > T (*n* = 1), 3849 + 10kbC > T/- (*n* = 1), N1303K/CFTRdele2,3(21kb) (*n* = 1), 1524 + 1G > A/3944delGT;406-6T > C (*n* = 1), 3272-26A > A/- (*n* = 1), none detected -/- (*n* = 3) [25,26]. The genotypes were classified by known clinical impact: severe/severe mutations (two class I-III mutations) and other mutations (at least one class IV-VI or unknown mutation) [27,28].

### 2.2. Sterols

Serum sterols were assessed using the method described previously by Corso et al. [29]. Blood samples were collected after overnight fasting, then 1N KOH in 90% ethanol (3 mL) was added to 50 µL serum spiked with 100 µL of an internal standard (5α-cholestane). Samples were hydrolysed for 60 min at 80 °C, then diluted with 3 mL of distilled water. Next, the samples were extracted three times using 2 mL of hexane. The upper phases were evaporated using nitrogen, then mixed in 100 µL of BSTFA and pyridine (7:3; v/v), before they were dried under nitrogen and redissolved in dichloromethane (100 µL). Samples (1 µL) were subjected to gas chromatography with mass spectrometry (GC-MS, Agilent 7890 series II and 5975C, Agilent Technologies, Santa Clara, USA) using the HP-5ms column (Agilent J&W GC Columns, Folsom, CA, USA). Peak integration was performed using MSD ChemStation (Agilent Technologies, Santa Clara, USA) and the concentrations of campesterol, β-sitosterol, stigmasterol and lathosterol were determined. The synthesis/absorption ratios (lathosterol/campesterol and lathosterol/β-sitosterol) were calculated to evaluate cholesterol metabolism. 

### 2.3. Lipid Profile 

Total serum cholesterol was measured by an enzymatic method employing cholesterol esterase, cholesterol oxidase and the Trinder reaction using the Integrated Chemistry System Analyser (Siemens Healthcare GmbH, Erlangen, Germany). Low-density lipoprotein cholesterol (LDL-C) and high-density lipoprotein cholesterol (HDL-C) concentrations were assessed directly using the catalase elimination method. 

### 2.4. Dietary Intake

The average daily intake of energy and lipids was calculated in 45 CF patients and 35 HS. The diet was analysed based on nutritional records collected via a 3-day diary (2 working days and one during the weekend; Dietetyk 2015 software; Jumar, Poznań, Poland). In CF patients, a standard dose of enzyme supplementation was calculated per gram of fat consumed. Regarding phytosterols, our own database was used based on a review of national and world literature of the content of phytosterols in food products of plant origin [30,31]. The NutritionData.com database was also applied in the study. 

### 2.5. Statistical Methods

The sterol concentrations are expressed as μg/dL of serum and the concentrations of TG, total cholesterol and its fractions are presented as mg/dL. For all parameters, medians and 1^st^–3^rd^ quartiles were calculated unless indicated otherwise. The Shapiro–Wilk test was used to check the normality of the data distribution, with the Mann–Whitney U-test or Fisher’s exact test used to assess differences between groups. The linear correlation between sterols intake and serum sterol concentrations (lathosterol and phytosterol/cholesterol and their fractions) was analysed using Spearman’s test. The relationship between the phytosterols, lathosterol and cholesterol and its fractions and all studied clinical parameters and nutritional intake was assessed using a multiple linear logistic regression (stepwise and backward). The following variables were interpreted as independent in all regression models: age, sex, BMI, *CFTR* genotype (severe/severe vs. other), FEV_1_, pancreatic insufficiency, dose of pancreatic enzymes (lipase units/gram of fat), liver disease, diabetes, *P. aeruginosa* colonisation. For cholesterol and its fractions, sterol concentrations were also included. A *p*-value <0.05 was considered statistically significant. In the graphic presentation, the relationship between the pancreatic enzyme dose and total cholesterol concentrations was smoothed using the LOWESS method. All statistical analyses were conducted using GraphPad Prism 5.01 (GraphPad Software, Inc., La Jolla, CA, USA) and Statistica 12.0 software (StatSoft Inc., Tulsa, USA).

### 2.6. Ethical Considerations

All participants gave their informed written consent for participation in the study. The study was approved by the Bioethical Committee of the Poznan University of Medical Sciences, Poznań, Poland (decision no 1225/16). The project was conducted in accordance with the Declaration of Helsinki.

## 3. Results

The anthropometric and clinical characteristics of the study groups are presented in Table 1. The CF patients had a significantly lower median of body height, weight and BMI than the HS group (*p* = 0.0022, *p* = 0.0295, *p* = 0.026, respectively).

**Figure 1:**
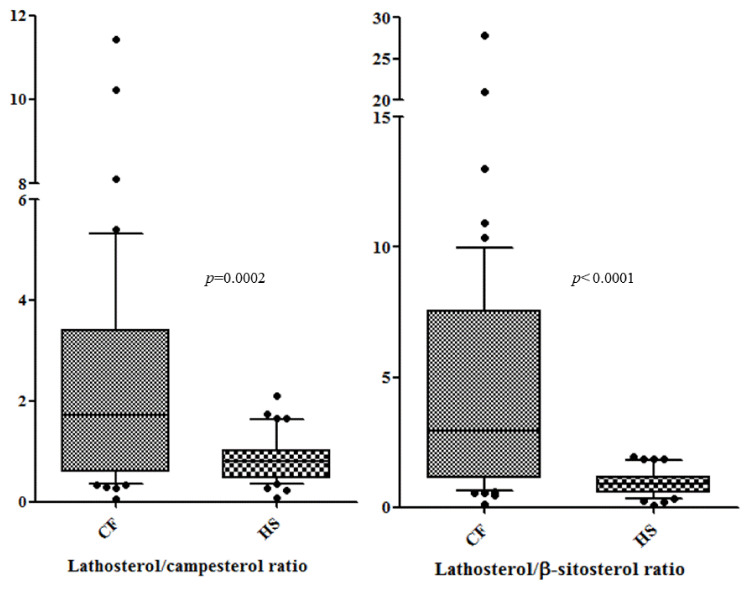
Lathosterol/campesterol and lathosterol/β-sitosterol ratios in cystic fibrosis (CF) patients and healthy subjects (HS). Box and whiskers: 10^th^–90^th^ percentiles and 1^st^–3^rd^ quartiles are shown.

Table 2 presents a comparison of all measured sterol concentrations between CF patients and HS. The concentrations of campesterol and β-sitosterol were lower in the CF patients (*p* = 0.0028 and *p* < 0.0001, respectively). The concentration of stigmasterol was comparable between analysed groups, whereas the level of lathosterol (a marker of endogenous cholesterol biosynthesis) was significantly higher in CF patients (*p* = 0.0016). Other sterols measured by the enzymatic methods were lower in CF patients (for total cholesterol *p* = 0.006, for HDL-C *p* < 0.0001, for LDL-C *p* = 0.0093). The synthesis/absorption ratios of sterols (lathosterol/campesterol and lathosterol/β-sitosterol) were higher in CF patients than HS (*p* = 0.0002 and *p*< 0.0001, respectively). The box plot of these ratios in CF patients and HS is shown in Figure 1.

The daily intake of energy (kcal), estimated energy requirement (%), total fat (g) and cholesterol (mg) intake were higher in CF patients than in HS (*p* = 0.0005, *p* = 0.0004, *p* = 0.0273, *p* = 0.0132, respectively). In 22 (40.0%) patients, the energy consumption was lower than the 110% of the RDA (Recommended Dietary Allowance), which is proposed as the minimum recommended intake for CF (independent of age, malnutrition or concurrent disease) [13]. Table 3 presents a comparison of all measured dietary intakes between CF patients and HS.

**Figure 2:**
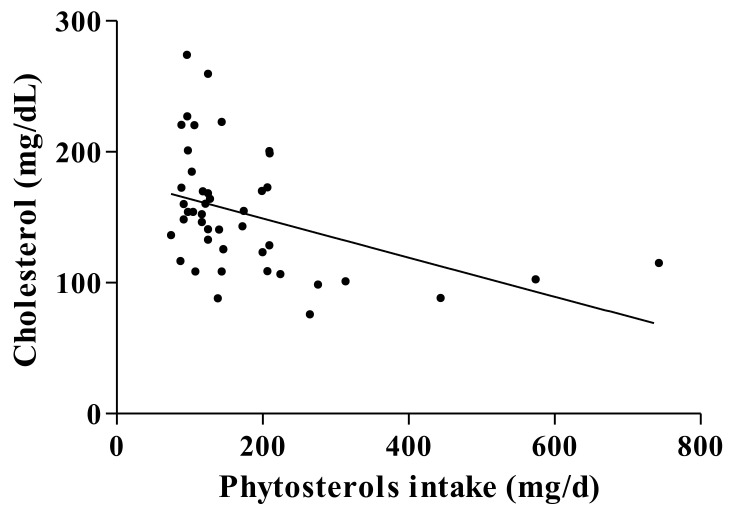
The relationship between phytosterols intake (mg/d) and serum cholesterol concentration (mg/dL) (*p* = 0.0007, r = -0.4888) in cystic fibrosis patients.

The correlation between total phytosterols intake and serum cholesterol concentration for CF patients was statistically significant (*p* = 0.0007, r = -0.4888; Figure 2).

The sterol concentrations in subgroups of CF patients defined by the different clinical parameters are presented in Table 4. Total cholesterol and lathosterol concentrations were lower in males. Lower campesterol, β-sitosterol, total cholesterol and LDL-C levels were documented in patients with a severe *CFTR* genotype and exocrine pancreatic insufficiency. Interestingly, the HDL-C concentration was lower only in patients with a severe *CFTR* genotype. Meanwhile, β-sitosterol concentrations were lower in patients colonised with *P. aeruginosa*. A lower lathosterol concentration was noted also in CF patients with poor lung function.

Forward stepwise regression revealed the following independent correlates of total cholesterol in CF: campesterol, age, sex, dose of pancreatic enzymes (lipase units/g of fat) and BMI; for HDL: age and sitosterol. Backward stepwise regression showed that the CFTR genotype and sitosterol were independent predictors of LDL concentrations (Table 5). The model seemed to explain a moderate fraction of the variability in the studied parameters.

Only one independent correlate of β-sitosterol (R^2^ = 0.2526, *p* = 0.002): CFTR genotype (β = −166.75, *p* = 0.00055), and campesterol (R^2^ = 0.2585, *p* = 0.0017): CFTR genotype (β = −192.17, *p* = 0.035), was identified.

**Figure 3:**
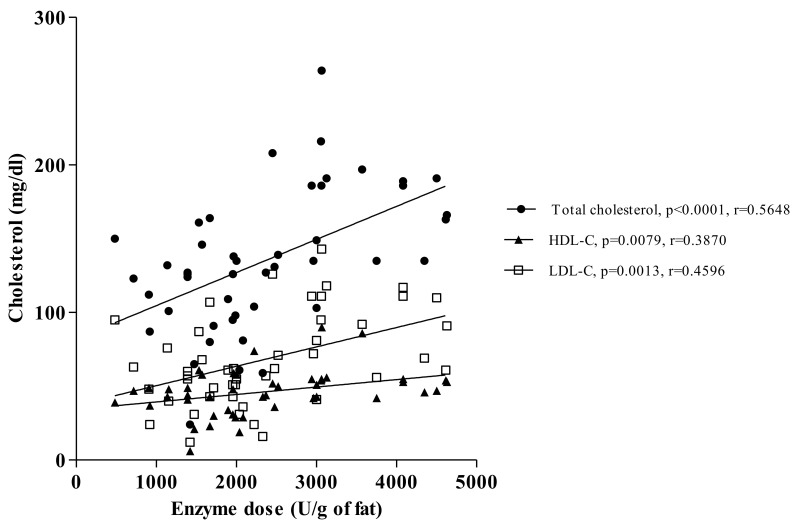
The relationships between enzyme intake (lipase units/g of fat) and serum cholesterol concentration (mg/dL) in pancreatic-insufficient patients.

The detailed analysis of pancreatic-insufficient patients revealed significant correlations between pancreatic enzyme dose (lipase units/g of fat) and serum cholesterol concentration and its fractions (*p* < 0.0001, r = 0.5648 for total cholesterol, *p* = 0.0079, r = 0.3870 for HDL-C and *p* = 0.0013, r = 0.4596 for LDL-C; Figure 3). 

**Figure 4:**
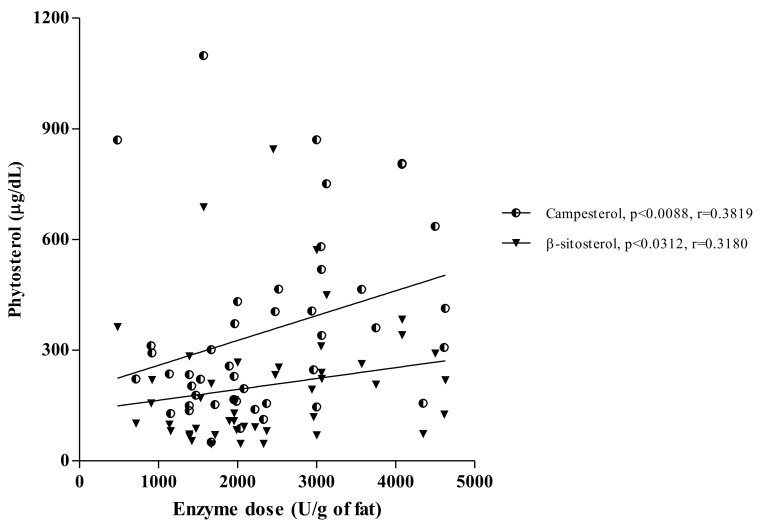
The relationships between enzyme intake (lipase units/g of fat) and phytosterol concentrations (μg/dL) in pancreatic-insufficient patients.

Similar associations were also documented between pancreatic enzyme dose (lipase units/g of fat) and phytosterol concentrations (*p* < 0.0088, r = 0.3819 for campesterol and *p* < 0.0312, r = 0.3180 for β-sitosterol), as presented in Figure 4.

**Figure 5:**
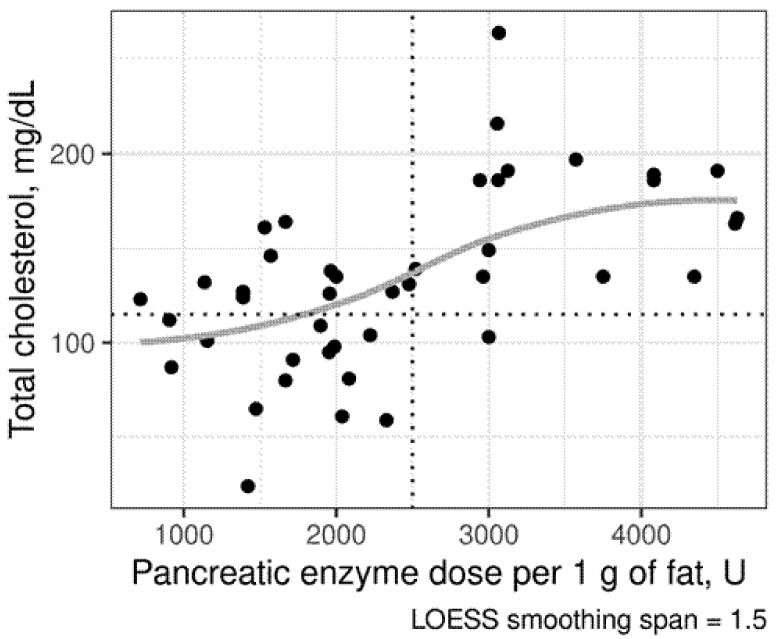
Scatterplot of total cholesterol in pancreatic-insufficient (PI) patients with cystic fibrosis (CF) with different doses of pancreatic enzymes. Points below the dotted horizontal line indicate patients with hypocholesterolemia as defined by the local laboratory reference range (< 115 mg/dL or 3 mmol/L). The vertical line was set at the enzyme dose of 2750 lipase units/g of fat, above which only one patient experienced hypocholesterolemia. The lower rate of hypocholesterolemia in PI CF patients receiving >2500 U/d of pancreatic enzyme replacement therapy was confirmed by Fisher’s test (two-sided *p* = 0.0026). Locally estimated scatterplot smoothing (LOWESS) curve is displayed in grey.

Subsequent analysis conducted in pancreatic-insufficient CF patients showed that the supplementation dose of 2500 U of lipase/g of fat was significant for hypocholesterolemia (Figure 5). The differences in sterol concentrations in subgroups receiving higher and lower doses are presented in Table 6.

**Figure 6:**
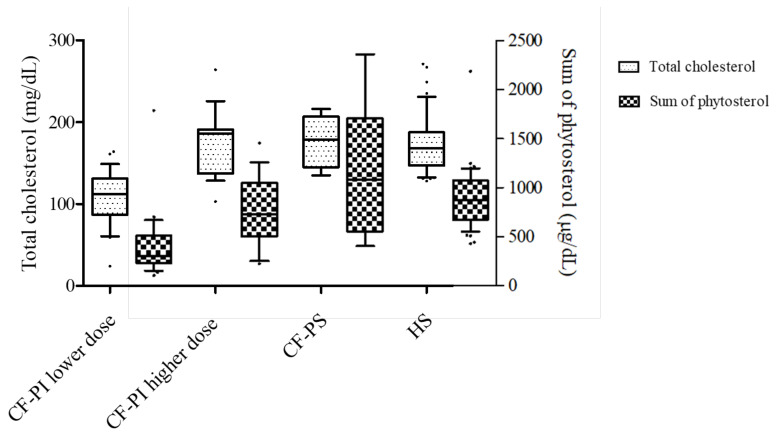
Sterol concentrations in cystic fibrosis patients: pancreatic-insufficient (CF-PI) subjects supplemented with lower (≤2500 U of lipase/g of fat) and higher (>2500 U of lipase/g of fat) doses of enzymes, pancreatic-sufficient (CF-PS) subjects and healthy subjects (HS). Total cholesterol (*) and sum of phytosterol (**) levels were higher for HS, CF-PS subjects and CF-PI patients supplemented with a higher dose of pancreatic enzymes than in the group CF-PI supplemented with a lower dose (*p* < 0.0001).

Total cholesterol and the sum of phytosterol concentrations in pancreatic-insufficient (PI) CF patients supplemented with a lower (≤2500 U of lipase/g of fat) and higher (>2500 U of lipase/g of fat) dose of pancreatic enzymes, pancreas-sufficient CF patients and HS are presented in Figure 6, showing that total cholesterol and the sum of phytosterol concentrations were significantly lower in the patients supplemented with a lower dose of the enzyme (*p* < 0.0001).

## 4. Discussion

This is the first study to assess lathosterol/campesterol and lathosterol/β-sitosterol ratios in CF with a concurrent analysis of sterols intake and dose of pancreatic enzymes. Higher concentrations of lathosterol and lower campesterol and β-sitosterol levels were noted in CF patients, indicating that the synthesis and absorption ratios were higher in CF patients than in HS. The daily intake of energy, fat and cholesterol was higher in CF patients than in HS; however, the daily intake of phytosterols was comparable. Serum total cholesterol and its fractions were lower in CF than in HS, which was related to phytosterol concentrations, age, dose of pancreatic enzymes, nutritional status and genotype. Furthermore, sitosterol and campesterol concentrations were predicted by genotype and pancreatic insufficiency, suggesting that the serum sterol profile in pancreatic-insufficient CF patients is strongly associated with the dose of pancreatic enzymes.

Hypocholesterolemia has been reported in CF, as evidenced by our previous analysis [7] and studies from other centres [32,33]. The reduction in total cholesterol and its fractions occurs in CF children, especially those with pancreatic insufficiency [34]. In our study, only seven (12.8%) CF patients were pancreatic-sufficient, so it is difficult to speculate about the impact of pancreatic sufficiency on the lipid profile. However, LDL-C concentrations were higher in pancreatic-sufficient than pancreatic-insufficient CF patients (Table 4). The multiple regression analysis identified the severe *CFTR* genotype as a predictor of lower total and LDL cholesterol concentrations. A study in a CF animal model showed that the exocytosis of cholesterol is impaired [10]. CFTR is a cAMP-activated chloride channel gated by the ATP, which structurally resembles the ATP-binding cassette (ABC). The ABCA1 protein controls the transport of cholesterol and phospholipids, presumably removing sterols from cells [35,36]. *CFTR* mutations trigger the cAMP pathway, leading to the inactivation of proteins involved in sterol exocytosis and, consequently, to the intracellular accumulation of cholesterol [37,38].

Our results show abnormalities in sterol homeostasis markers. Lathosterol is the most reliable surrogate marker of de novo liver cholesterol synthesis [39], which can be produced in other tissues but does not pass into the bloodstream; hence, it is considered to be liver-specific [40,41]. Higher lathosterol concentrations may be directly related to *CFTR* mutations. The in vitro data showed that a *CFTR* mutation might influence cholesterol trafficking to the plasma membrane and is associated with an increased expression of the cholesterol transport protein NPC1 in CF cells [42]. Fang et al. also reported that CFTR influences cholesterol trafficking to the plasma membrane, which, when depleted, leads to an increase in de novo cholesterol synthesis to restore the membrane content [9].

Campesterol and sitosterol were measured in our study as surrogate markers of sterol absorption [43] and their increased malabsorption was confirmed. We documented low phytosterol concentrations in CF patients with a severe *CFTR* genotype and exocrine pancreatic insufficiency. The association between low sterols and severe *CFTR* is in agreement with the very high grade of pancreatic insufficiency in CF patients with the F508del homozygous genotype [44]. Gelzo et al. suggested that pancreatic insufficiency cannot be considered as the only and sufficient explanation of decreased phytosterol levels in CF [18,19]; however, they did not perform additional analysis to confirm their suggestion. Reduced absorption of sterols could be caused by liver disease and the impaired secretion of biliary salts, which are essential cofactors of cholesterol esterase that helps to absorb cholesterol [45]. However, our study did not confirm the effect of liver disease on the observed lipid disturbances, indicating the association with the dose of pancreatic enzymes. Further, other factors should be considered, such as the lower (negligible) cholesterol esterase activity of commercially available (pig) enzymes [42], intestinal mucosal abnormalities, lower alkaline duodenal pH, changed bile salt profile or small intestinal bacterial overgrowth, which are present in a significant percentage (35–37%) of CF patients [45,46,47,48,49]. Interestingly, in the present study, multiple regression analyses showed that campesterol and β-sitosterol levels were predictors of high cholesterol and its fractions in patients with CF. However, CF dyslipidemia comprises hypocholesterolemia, predominantly in PI patients [7]. Serum sterol concentrations at diagnosis in a CF neonatal screening programme could be an interesting perspective for future studies to define pancreatic status [50].

Standard CF therapy includes a high-energy, high-fat diet (with pancreatic enzyme replacement therapy) [13,51], and it is recommended to use good-quality fats comprising polyunsaturated fatty acids from oils, nuts and avocados (products that are also a source of plant sterols) [13,30,52]. Regardless of the guidelines, the phytosterol intake in the present study was comparable in CF patients and HS as a result of the high consumption of saturated fatty acids, butter, eggs and dairy, which was also documented by our previous research [53]. Without any doubt, nutritional recommendations demand individualization. Some patients may have the same energy requirements as healthy peers with no need for a high fat intake. The particular emphasis should be put on the qualitative aspect of the diet comprising, among others, the determination of the fatty acid profile [13,54,55]. 

The main limitation of this study is its cross-sectional character, which makes inferring causality impossible. Moreover, the reliability of food diaries always, even in highly educated CF patients, raises some doubts; however, we aimed to maximise the precision of the nutritional assessment through education and communication with the study participants. The estimation of the phytosterol content of foods is also challenging, so we created a database from various information sources.

## 5. Conclusions

Endogenous liver production of cholesterol is increased, whereas intestinal sterol absorption is decreased in CF patients. Independent determinants of hypocholesterolemia and low levels of campesterol and β-sitosterol suggest that malabsorption may play a significant role in sterol abnormalities. A better serum sterol profile in pancreatic-insufficient patients is associated with high doses of pancreatic enzymes. Therefore, the measurement of campesterol and β-sitosterol concentrations in CF patients may potentially serve for the assessment of the effectiveness of pancreatic enzyme replacement therapy and/or compliance with the given recommendations, but further research is required.

## Figures and Tables

**Table 1:** Anthropometric and clinical data of cystic fibrosis (CF) patients and healthy subjects (HS).

Parameters	CF Patients	HS	*P*
	Median (1st–3rd quartile)		
N	55	45	−–
Age [years]	26.9 (20.5–32.0)	25.4 (22.4–28.3)	ns.
Sex ratio [F/M]	33/22 (60.0%)	26/19 (57.8%)	ns.
Body height [m]	1.69 (1.6–1.7)	1.71 (1.6–1.8)	0.0295
Body weight [kg]	57.0 (51.4–65.0)	65.0 (57.2–75.5)	0.0022
BMI [kg/m^2^]	20.5 (18.7–22.8)	21.9 (20.1–24.0)	0.0260
Pancreatic insufficiency	87.2%	−	−
FEV1 [%]	56.0 (40.0–81.0)	−	−

BMI—body mass index. FEV1—forced expiratory volume in one second.

**Table 2:** Sterols’ concentrations in cystic fibrosis (CF) patients and healthy subjects (HS).

Method	Clinical Parameters	CF Patients	HS	*p*
		Median (1st–3rd Quartile)		
Chromatographic	Campesterol [μg/dL]	292 (172–492)	452 (363–514)	0.0028
	β-sitosterol [μg/dL]	165 (92–283)	401 (319–507)	<0.0001
	Stigmasterol [μg/dL]	9 (7–24)	14 (12–19)	ns.
	Lathosterol [μg/dL]	480 (281–720)	286 (235–435)	0.0016
Enzymatic	Total cholesterol [mg/dL]	135 (110–176)	168 (147–185)	0.0006
	HDL-C [mg/dL]	49 (42–56)	66 (53–67)	<0.0001
	LDL-C [mg/dL]	66 (51–94)	83 (75–105)	0.0093
Ratio	Lathosterol/Campesterol	1.73 (0.64–3.43)	0.80 (0.48–0.99)	0.0002
	Lathosterol/β-sitosterol	2.93 (1.18–6.62)	0.91 (0.54–1.15)	<0.0001

HDL-C—high-density lipoprotein cholesterol. LDL-C—low-density lipoprotein cholesterol.

**Table 3:** Dietary intakes in cystic fibrosis (CF) patients and healthy subjects (HS).

Parameters	CF Patients	HS	*p*
	Median (1st–3rd Quartile)		
N	45	35	—
Energy [kcal/d]	2433 (2114–2699)	1850 (1730–2245)	0.0005
EER [%]	121.5 (96.8–130.5)	94.4 (86.6–101.2)	0.0004
Total fat [% en]	37.3 (33.5–40.5)	39.1 (33.9–45.0)	ns.
Total fat [g/d]	102.2 (86.4–119.5)	84.3 (71.3–107.5)	0.0273
Cholesterol [mg/d]	332.4 (244.6–435.0)	288.2 (149.1–315.3)	0.0132
Total phytosterol [mg/d]	125.2 (101.5–198.8)	151.3 (131.1–214.8)	ns.
Campesterol [mg/d]	28.5 (22.6–44.6)	33.7 (25.6–48.8)	ns.
Β-sitosterol [mg/d]	72.2 (57.1–112.5)	88.8 (70.9–145.1)	ns.
Stigmasterol [mg/d]	3.1 (1.8–4.5)	4.0 (3.0–6.9)	ns.
Lipase dose * [U/g of fat]	2060 (1540–3042)	—	—

EER—estimated energy requirement. *—for pancreatic-insufficient patients.

**Table 4:** Sterol concentrations in subgroups of cystic fibrosis patients defined by different clinical parameters.

Parameters	Sex		BMI [kg/m^2^]		*CFTR* Genotype		Exocrine Pancreatic Insufficiency		Liver Disease		Diabetes		*P. aeruginosa*		FEV1 [%]	
Median 1^st^–3^rd^ Quartiles	Women	Men	≤21	>21	Severe	Other	Yes	No	Yes	No	Yes	No	Yes	No	<80	>80
N	33	22	28	27	32	23	48	7	26	29	9	46	37	18	39	16
Campesterol [μg/dL]	298.9 207.6–505.5	356.8 152.5–465.1	274.9 175.0–411.1	298.9 223.9–565.2	225.6 *** **160.2–308.3**	549.8 *** **311.1–790.6**	256.8 ** **163.8–422.3**	733.7 ** **479.9–1015.0**	251.8 157.6–402.8	309.5 227.2–599.2	221.9 166.3–292.4	307.1 195.6–587.2	257.3 166.3–431.3	360.7 221.9–587.2	301.5 199.4–492.0	257.3 152.6–52.0
β–sitosterol [μg/dL]	167.9 97.3–296.5	156.2 87.4–283.6	159.5 86.4–241.5	20.1.6 97.3–289.4	108.3 *** **81.1–210.2**	273.3 *** **172.4–376.8**	156.2 ** **85.5–246.1**	362.7 ** **244.7–520.1**	113.6 81.7–221.4	214.6 114.8–285.5	160.1 101.7–170.3	193.5 91.8–291.3	126.1 * **87.4–267.1**	207.4 * **156.2–283.6**	165.4 95.6–265.1	159.1 86.4–297,3
Stigmasterol [μg/dL]	10.4 7.7–23.9	9.1 7.0–23.7	12.7 8.5–24.1	8.2 7.2–23.5	9.0 7.2–23.5	9.8 7.6–23.7	8.4 7.2–23.0	20.9 9.8–27.6	9.2 7.5–20.9	8.8 7.2–23.8	5.9 5.0–6.8	9.2 7.4–23.8	12.1 8.4–24.6	7.8 6.2–9.5	9.1 7.1–23.0	8.4 7.8–24.5
Lathosterol [μg/dL]	543.3 * **414.8–814.2**	381.7 * **253.3–531.5**	448.3 242.5–618.7	569.3 388.9–790.1	489.7 263.0–735.4	476.5 260.9–704.8	486.5 300.1–739.4	472.8 287.7–506.1	505.8 292.9–722.7	480.1 317.1–717.5	314.6 199.1–468.5	493.0 372.1–731.5	480.2 257.0–696.4	493.0 372.1–819.1	432.9 * **258.8–604.4**	696.4 * **510.1–814.3**
Total cholesterol [mg/dL]	143.0 * **131.0–182.0**	123.0 * **91.0–166.0**	135.0 97.2–152.0	157.0 123.2–186.0	125.0 *** **94.0–136.0**	184.0 *** **144.5–195.5**	135.0 * **103.5–167.5**	182.0 * **146.5–205.5**	135.0 98.7–158.2	144.0 125.5–186.0	123.0 98.0–161.0	135.0 124.0–186.0	135.0 101.0–166.0	161.0 124.0–186.0	135.0 105.0–172.0	135.0 126.0–184.0
HDL–C [mg/dL]	51.0 43.0–57.0	47.0 37.8–55.3	45.0 34.8–58.0	49.5 44.0–55.0	44.5 ** **33.3–51.5**	54.5 ** **48.3–57.8**	48.0 41.5–55.0	53.0 50.5–57.5	46.5 35.3–55.5	49.5 43.0–55.5	48.0 37.0–59.0	49.0 42.0–55.5	48.0 36.0–54.0	51.0 47.0–56.0	47.0 36.5–56.0	51.5 47.0–58.5
LDL–C [mg/dL]	71.0 57.0–107.0	57.5 44.3–91.3	61.5 50.3–74.3	90.5 56.3–109.8	55.0 *** **42.5–69.5**	95.0 *** **76.0–111.0**	62.0 ** **48.0–87.0**	109.0 ** **95.0–126.0**	62.0 51.0–89.3	76.0 55.0–109.0	62.0 51.0–83.0	70.0 55.0–104.0	62.0 49.0–91.0	82.0 57.8–109.3	68.0 51.0–93.5	65.5 52.8–95.0

* *p*<0.05; ** *p*<0.01; *** *p*<0.001; HDL–C–high–density lipoprotein cholesterol; LDL–C–low–density lipoprotein cholesterol.

**Table 5:** Forward (total cholesterol and HDL) and backward (LDL) stepwise regression analyses of various factors predictive of cholesterol and its fractions in cystic fibrosis patients.

Dependent Variable	*p* Model	R^2^	Independent Variables	β	*p*
Total cholesterol	10^−6^	0.6242	Campesterol	0.0686	0.000046
			Age	2.0042	0.0011
			Sex	26.1551	0.0048
			U of lipase/g of fat	0.0073	0.032
			BMI	3.1605	0.040
HDL-C	0.0143	0.3841	Age	0.63768	0.025
			β-sitosterol	0.04741	0.048
LDL-C	10^−6^	0.5075	CFTR genotype	−29.5117	0.00011
			β-sitosterol	0.0665	0.0016

HDL-C—high-density lipoprotein cholesterol; LDL-C—low-density lipoprotein cholesterol.

**Table 6:** Sterol concentrations in subgroups of cystic fibrosis patients supplemented with higher and lower pancreatic enzyme doses.

Enzyme Dose [Lipase units/g of Fat]	≤2500	>2500	*P*
	Median (1st–3rd Quartile)		
N	28	17	−
Campesterol [μg/dL]	221.1 (152.5–301.5)	465.1 (340.0–635.4)	0.0014
β-sitosterol [μg/dL]	101.7 (81.0–209.7)	238.9 (193.5–311.0)	0.0078
Stigmasterol [μg/dL]	9.2 (8.3–23.5)	7.5 (7.1–12.1)	ns.
Total cholesterol [mg/dL]	123.0 (91.0–132.2)	186.1 (139.2–191.0)	<0.0001
Lathosterol [μg/dL]	486.5 (314.6–707.5)	480.2 (261.4–761.8)	ns.

## Data Availability

Data shared in this manuscript are in accordance with consent provided by participants on the use of confidential data. The data presented in this study are available within this article.
